# Psychosocial mechanisms of sustained learning engagement in university Tai Chi courses: a qualitative study of teacher–peer dual-support synergy and learning pathways

**DOI:** 10.3389/fpubh.2026.1876692

**Published:** 2026-09-15

**Authors:** Lan Li, Qianqian Fan

**Affiliations:** Physical Education Department, Huanggang Normal University, Huanggang, China

**Keywords:** embodied learning, learning engagement, motivational internalization, psychosocial mechanisms, self-efficacy, social support

## Abstract

**Background:**

In university physical education courses, Tai Chi, as a course requiring a high level of bodily coordination and sustained practice, poses challenges in maintaining students’ learning engagement. Although teacher support and peer support are widely regarded as important psychosocial resources for promoting learning engagement, their mechanisms of action and interactive processes in skill-based embodied learning contexts remain insufficiently explained. In particular, how social support facilitates sustained learning engagement through psychological mechanisms has not yet been fully clarified.

**Methods:**

This study employed a descriptive and inductive qualitative research design, conducting semi-structured interviews with teachers (*N* = 8) and students (*N* = 16) in university Tai Chi courses. Credibility was enhanced through independent coding, negative case analysis, and reflexive memoing. Data analysis combined thematic analysis and the constant comparative method, using a systematic process of open, axial, and selective coding. This process ultimately led to the construction of a theoretical framework explaining the mechanism underlying the formation of sustained learning engagement.

**Results:**

The study identified three integrated psychosocial pathways: (1) teaching empowerment: teachers provided structured support through layered demonstration, protective feedback, and the construction of cultural meaning, thereby facilitating students’ mastery experiences and strengthening self-efficacy; (2) motivational internalization: peers, through emotional companionship, behavioral modeling, and collaborative responsibility, gradually transformed external participation into internal value endorsement; (3) dual-support synergy: teacher support and peer support interacted and reinforced each other throughout the learning process, jointly sustaining learning engagement. In addition, negative cases further revealed boundary conditions under which the support system failed.

**Conclusion:**

The “dual-support–dual-pathway model” proposed in this study indicates that sustained learning engagement in embodied learning contexts is the result of the combined effects of self-efficacy accumulation generated through teaching empowerment and motivational internalization facilitated by peer interaction. These two pathways can operate relatively independently, while also forming synergistic reinforcement within a psychosocial system. This study extends the understanding of the mechanisms underlying the formation of learning engagement and provides an integrated psychosocial explanatory framework for learning engagement in embodied learning contexts.

## 1 Background

University physical education courses play an important role in promoting students’ physical and mental health and cultivating long-term healthy lifestyles. In recent years, higher education institutions have not only emphasized students’ short-term physical activity participation in classroom settings but have also increasingly highlighted the sustained maintenance of health-related behaviors beyond the classroom (1). Among university physical education programs, Tai Chi has attracted growing attention in the fields of physical education and health promotion due to its characteristics involving bodily movements, breathing regulation, attentional control, and mind–body coordination (2). As a form of physical activity with distinctive traditional Chinese cultural features, Tai Chi is considered beneficial for stress regulation, emotional improvement, and long-term health promotion (3). However, for university students without basic training, learning Tai Chi represents a challenging skill-based activity. Its slow rhythm and complex structure place high demands on bodily coordination and sustained practice, making students prone to frustration, anxiety, and declining motivation during the learning process (4). Therefore, how to maintain sustained learning engagement in university Tai Chi courses has become an important issue in both higher education physical education and public health practice.

Learning engagement refers to students’ behavioral participation, emotional involvement, and cognitive concentration in learning activities (5). Existing research indicates that sustained learning engagement is not only closely associated with skill acquisition and learning persistence, but also strongly linked to individuals’ self-regulation abilities and levels of psychological wellbeing (6). In the context of physical activity, sustained engagement is particularly important, as it may influence whether students continue to maintain health-promoting exercise behaviors after the course ends (7). Health promotion research emphasizes the role of supportive educational environments in facilitating the maintenance of health behaviors, suggesting that a positive social support environment can serve as an important psychosocial resource that helps young people develop sustained health-related engagement (8). In university physical education courses, social support is widely recognized as a key external factor influencing students’ learning motivation and physical activity participation (9).

Nowadays, college students generally face heavy academic stress, declining psychological wellbeing and weak long-term exercise motivation, which leads to superficial participation in physical education. Tai Chi, as a mind–body integrated embodied sport, can ease learning anxiety, build movement confidence and cultivate group belonging, so it serves as a targeted intervention to solve the disconnection between short classroom participation and sustained health exercise.

Teacher support and peer support are the two most prominent forms of social support in educational contexts. Teacher support typically includes instructional guidance, evaluative feedback, emotional encouragement, and the creation of a psychologically safe learning environment (10). In skill-based learning processes, teachers can help students accumulate successful experiences, reduce learning anxiety, and enhance learning confidence through task scaffolding, step-by-step demonstration, and supportive feedback (11, 12). In contrast, peer support is more embedded in horizontal interactions among students, and is mainly reflected in emotional companionship, collaborative practice, social comparison, and a sense of shared responsibility (13). Through peer interaction, students may gradually transform participation initially driven by course requirements into autonomous engagement characterized by personal meaning and social identification (14). Although existing studies have separately confirmed the important effects of either teacher support or peer support on learning engagement (15), there is still a lack of research that situates both within a unified framework and explores their synergistic mechanisms in skill-based physical education contexts, particularly in Tai Chi courses that heavily rely on embodied perception and sustained practice.

Self-efficacy and motivational internalization may be two key psychological mechanisms for understanding how social support promotes sustained learning engagement. Self-efficacy theory posits that individuals develop the belief that “I am capable of completing tasks” through mastery experiences, verbal persuasion, and vicarious experiences, and this belief in turn influences behavioral persistence and effort levels (11, 12). In the process of learning Tai Chi, structured guidance and supportive feedback provided by teachers may help students develop a stable sense of efficacy regarding their physical abilities and movement mastery (16). In addition, when individuals experience a sense of competence, relatedness, and supportive interpersonal relationships during learning, behaviors originally driven by external factors may gradually be internalized into more autonomous forms of motivation (14). Therefore, in collective Tai Chi practice, emotional companionship and group belonging among peers may encourage students to shift from “completing requirements” to “actively identifying with” sustained engagement. However, there is still a lack of in-depth qualitative research grounded in authentic teaching contexts on how teacher support and peer support jointly influence sustained learning engagement through these psychological mechanisms. Moreover, existing studies have largely examined teacher support and peer support as separate influences and have relied predominantly on quantitative designs, leaving limited understanding of how these forms of support interact and operate through psychosocial processes in embodied learning contexts such as university Tai Chi courses. Furthermore, existing research has typically applied self-efficacy, self-determination, and health-promotion perspectives in isolation. Consequently, limited attention has been given to how these complementary processes jointly explain sustained learning engagement in embodied learning contexts.

Current research on university students’ physical activity and mental health has predominantly adopted quantitative methods. Although these studies can demonstrate statistical relationships between variables, they are limited in explaining the contextualized nature and individual experiential characteristics of social support within real teaching interactions (17, 18). In contrast, qualitative research can systematically elucidate how students interpret supportive experiences, cope with learning difficulties, and develop sustained learning engagement through everyday interactions (19, 20). Tai Chi, in particular, relies heavily on the interplay among embodied perception, emotional regulation, and interpersonal interaction throughout the learning process. Therefore, this issue is especially salient in the context of Tai Chi courses.

Based on this, the present study focuses on university Tai Chi courses to examine how teacher support and peer support promote students’ sustained learning engagement. Through semi-structured interviews with university teachers and students, this study aims to construct a process-oriented framework for understanding how social support facilitates sustained learning engagement, thereby illustrating the psychosocial pathways underlying the formation of learning engagement in skill-based physical activity contexts. Accordingly, this study addresses the following research question:

How do teacher support and peer support in university Tai Chi courses promote students’ sustained learning engagement, and through what psychosocial processes do students develop self-efficacy, motivational internalization, and continued participation?

## 2 Methods

This study employed a descriptive and inductive qualitative research design, using the thematic analysis approach proposed by Braun and Clarke (21) to explore how teacher support and peer support promote students’ sustained learning engagement in university Tai Chi courses. Thematic analysis is well suited for examining participants’ experiences, meaning-making processes, and interactional dynamics within specific social contexts, enabling researchers to identify patterns and themes relevant to the research questions from rich narrative accounts (22). At the same time, an inductive, data-driven analytical approach was adopted, with a focus on how students and teachers understand, experience, and sustain learning engagement, rather than testing an *a priori* theoretical framework.

### 2.1 Study setting

This study was conducted in university Tai Chi courses at two comprehensive higher education institutions in China. Tai Chi was selected as the research context because it represents a typical skill-based physical activity characterized by complex movement structures, a long learning cycle, and a strong reliance on sustained practice, bodily coordination, and interpersonal interaction (23). Compared with general physical education courses, Tai Chi not only emphasizes movement mastery but also highlights breathing regulation, bodily awareness, and mental focus; therefore, it is particularly suitable for examining how social support influences students’ sustained learning engagement. All courses were implemented according to the standard academic semester schedule of the universities. Teachers conducted instruction based on a unified syllabus while retaining a degree of instructional autonomy, including classroom organization, practice arrangements, and feedback strategies. This naturalistic educational setting provided an appropriate context for observing the relationships among teacher support, peer interaction, and students’ learning experiences.

### 2.2 Participants and sampling

The study employed purposive sampling to recruit participants, ensuring that they could provide rich and diverse experiences of learning and teaching (24). The inclusion criteria were as follows: (1) currently enrolled in a university Tai Chi course; (2) aged 18 years or older; (3) willing to share their learning or teaching experiences; and (4) for teacher participants, at least one full semester of teaching experience in university Tai Chi courses. In addition, a maximum-variation sampling strategy was applied to ensure representation of different levels of teaching experience, disciplinary backgrounds, and academic ranks among teachers, as well as different academic years and majors among students. A total of 24 participants were selected, including eight teachers and 16 students. Recruitment continued until thematic saturation was achieved, meaning that no substantially new themes or conceptual insights emerged from subsequent interviews. Saturation was assessed through ongoing comparison of interview data during analysis. Teacher participants varied in teaching experience, disciplinary backgrounds, and academic ranks, thereby enhancing the diversity of instructional perspectives (Table 1). Student participants were drawn from different academic years and majors to capture learning experiences across varied participant roles (Table 2; full details are provided in Appendices S1, S2).

**Table 1:** Teacher participant profiles (*n* = 8).

ID	Gender	Years of teaching	Education	Discipline	Academic rank	Key background (condensed)
T01	Male	10	Master’s	Traditional Chinese Physical Education	Lecturer	Martial arts teaching background; emphasizes movement decomposition and bodily awareness
T02	Female	18	Bachelor’s	Physical Education	Associate Professor	Focuses on supportive classroom climate and individualized encouragement
T03	Male	5	Master’s	Exercise Physiology	Teaching Assistant	Integrates physiological understanding into Tai Chi instruction
T04	Female	12	Master’s	Physical Education Training	Lecturer	Emphasizes movement precision and post-class practice guidance
T05	Male	22	Bachelor’s	Traditional Chinese Medicine	Professor	Connects Tai Chi practice with traditional health concepts
T06	Female	7	Master’s	Psychology	Lecturer	Focuses on motivation, confidence, and collaborative learning
T07	Male	15	Doctorate	Sociology of Sport	Associate Professor	Emphasizes peer interaction and embodied learning experiences
T08	Female	3	Master’s	Martial Arts and Ethnic Sports	Teaching Assistant	Uses multimedia demonstrations and peer examples in teaching

**Table 2:** Student participant profiles (*n* = 16).

ID	Gender	Academic year	Major category	Role/experience	Key background (condensed)
S01	Male	Freshman	Engineering	None	No prior Tai Chi experience; relied heavily on teacher guidance
S02	Female	Freshman	Economics and Management	Class Arts and Sports Committee Member	Actively participated in peer practice activities
S03	Male	Sophomore	Agricultural Sciences	Tai Chi Club Officer	Demonstrated relatively high movement confidence
S04	Female	Sophomore	Medicine	None	Experienced difficulty memorizing movement sequences
S05	Male	Sophomore	Information Technology	None	Sensitive to rhythm and peer encouragement
S06	Female	Junior	Teacher Education	Student Union Officer	Used Tai Chi practice for stress regulation
S07	Male	Junior	Engineering	None	Preferred collaborative and practice-oriented learning
S08	Female	Freshman	Arts	Psychological Committee Member	Initially lacked confidence in bodily coordination
S09	Male	Sophomore	Mechanical Engineering	Group Leader	Developed confidence through leading group practice
S10	Female	Junior	Economics and Management	Intern	Participated in collective practice during internship period
S11	Male	Freshman	Automotive Engineering	None	Viewed teacher as an important behavioral role model
S12	Female	Junior	Information Technology	Peer Teaching Assistant	Strengthened confidence through assisting peers
S13	Male	Sophomore	Medicine	None	Initially resistant but gradually adapted through peer companionship
S14	Female	Freshman	Agriculture and Forestry	Dormitory Life Committee Member	Transitioned gradually from observer to participant
S15	Male	Junior	Civil Engineering	Club Leader	Demonstrated strong engagement during group performances
S16	Female	Sophomore	Arts	None	Highly responsive to teachers’ supportive feedback

### 2.3 Data collection

Data were collected through semi-structured interviews. The interview guide was designed around key themes including teacher support, peer support, self-efficacy, learning motivation, and learning engagement (full protocol provided in Appendix S3). Sample questions included: “How do you help students with poor coordination build confidence?,” “What teaching or peer interactions most influenced your willingness to continue practicing?,” “How did your confidence or motivation change throughout the course?,” and “How do you understand the value of Tai Chi beyond course requirements?” To avoid capturing only positive experiences, questions regarding negative and boundary experiences were also included, such as: “Have you ever experienced negative peer comparison?” and “Are there any teaching experiences that made you unwilling to continue practicing?” This was intended to enhance the completeness and credibility of the findings.

Participants were recruited through course coordinators and referral sampling. All interviews were conducted face-to-face in quiet classrooms, with each session lasting approximately 30–45 min. Prior to the interviews, participants were informed of the study purpose, confidentiality principles, and their right to withdraw, and written informed consent was obtained. All interviews were audio-recorded with participants’ permission and transcribed verbatim. The total interview data amounted to approximately 51,000 Chinese characters. Data were anonymized using participant codes (T01–T08 for teachers; S01–S16 for students), and all personally identifiable information was removed.

### 2.4 Analysis

Data analysis was managed and organized using NVivo 14 software and followed the six-phase thematic analysis process (21, 22): (1) Familiarization with the data: the researchers repeatedly read all interview transcripts to develop a holistic understanding of the data and its context, while recording initial analytical memos, such as recurring experiential patterns including “teachers’ step-by-step demonstration,” “emotional companionship from peers,” and “changes from tension to relaxation during learning”; (2) Initial coding: a line-by-line open coding approach was applied to systematically code the entire dataset, focusing on experiences related to teacher support, peer support, learning motivation, self-efficacy, and sustained engagement. Both positive experiences and negative/boundary cases were retained to avoid an overly positivity-biased analysis; (3) Category development: initial codes were grouped and organized based on conceptual similarity and relational connections, gradually forming higher-order analytical categories. This stage primarily focused on teacher-led structured support, peer interaction experiences, the process of efficacy development, and changes in learning engagement; (4) Theme review and refinement: candidate themes were repeatedly compared and refined through ongoing discussion, with attention to internal coherence and external distinctiveness between themes. Negative and boundary cases were retained to test thematic boundaries, thereby enhancing the credibility and interpretive robustness of the analysis; (5) Theme definition and naming: based on the preceding analysis, three integrated themes were ultimately identified: (a) teaching empowerment pathway; (b) peer internalization pathway; and (c) dual-synergy pathway. Together, these themes illustrate how teacher support and peer support promote sustained learning engagement through distinct psychosocial mechanisms; (6) analytic writing: the relationships among themes were synthesized into a coherent analytical narrative, supported by representative participant quotations to reflect the experiential grounding and contextual meaning of each theme. The full coding structure and theme development process are provided in Appendices S4–S7. For example, statements such as “the teacher broke movements into smaller parts” were initially coded as instructional decomposition and later incorporated into the broader category of cognitive scaffolding within the teaching empowerment pathway.

### 2.5 Researcher positionality and trustworthiness

The researchers had experience in Tai Chi practice and in observing university physical education teaching, which enabled a strong understanding of instructional interactions and student experiences within the research context. However, the researchers also recognized that prior familiarity with Tai Chi and university physical education might influence interactions with participants and the interpretation of the data. To minimize potential bias, reflexive journals were maintained throughout the research process to document preconceptions, emotional responses, and analytical decisions during data collection and analysis, while particular attention was paid to critically examining alternative interpretations and negative experiences reported by participants (22). In addition, multiple strategies were employed to enhance research trustworthiness (25). Credibility was strengthened through independent coding, theme discussions, and the retention of negative and boundary cases to examine the stability of interpretations. Dependability was supported through systematic documentation of coding decisions and analytical procedures throughout the research process. Confirmability was enhanced through reflexive journaling and researcher discussions aimed at minimizing subjective bias. Transferability was facilitated through thick descriptions of the educational context and the use of representative participant quotations to allow readers to assess the applicability of the findings to other settings.

In addition, all data were anonymized, and strict informed consent and confidentiality procedures were followed. We further reflected on power gaps in interviews: our academic identity formed implicit hierarchy with student interviewees. To reduce bias brought by our prior Tai Chi and PE research experience, we used neutral open-ended questions and cross-examined all interview narratives with negative boundary cases during data coding and interpretation. The reporting of this study was informed by the Consolidated Criteria for Reporting Qualitative Research.

## 3 Results

The results of this study are organized around three integrated themes that describe the process through which teacher support and peer support influence students’ sustained learning engagement in university Tai Chi courses. Participants’ experiences revealed a developmental trajectory shifting from “external support” to “internal motivation.” Theme 1 describes how teachers foster students’ confidence in “I can do it” through structured instructional support. Theme 2 illustrates how peer interaction promotes the gradual internalization of learning motivation through emotional companionship and collaborative relationships. Theme 3 presents the intertwined and synergistic functioning of teacher support and peer support within the actual teaching process.

### 3.1 Theme 1: instructional empowerment pathway

Theme 1 describes how teachers, through systematic instructional support, help students gradually move from an initial state of unfamiliarity, tension, and uncertainty toward perceivable mastery experiences, ultimately fostering a stable sense of self-efficacy. This pathway is particularly evident among beginners and students with lower levels of bodily coordination.

#### 3.1.1 Cognitive scaffolding and psychological safety

Students noted that teachers reduced the difficulty of learning Tai Chi by providing step-by-step demonstrations, breaking down movements, and adopting flexible practice standards, enabling them to gradually complete movement sequences that initially appeared complex. “At the beginning, I could not even remember four movements. The teacher broke the routine down very clearly. When I successfully completed the first segment, I started to feel that maybe I could really learn this” (S01). Teachers described similar instructional strategies: “I break one movement into three smaller parts. Once students realize they can follow the first part, they gain confidence to proceed to the second” (T01). In addition to technical guidance, the non-judgmental classroom atmosphere created by teachers was frequently emphasized by students. Students reported that when they perceived teachers’ patience and tolerance, they were more willing to attempt movements and expose their shortcomings without fear of being laughed at. “The teacher’s calm tone and patient step-by-step guidance created a sense of psychological safety, allowing me to perform clumsily without feeling embarrassed” (S01). Teachers also observed that some students, although technically capable of performing movements, were hindered by excessive tension and hesitation. “Sometimes I see that her movements are actually fine, but she is too nervous. I walk over, gently pat her shoulder, and say: you are doing well, do not rush” (T02). In addition, negative cases indicated that when teachers overemphasized movement accuracy, students tended to experience practice-related anxiety (b1), whereas students who lacked sustained individual attention were more likely to withdraw to the back of the class and maintain only superficial participation (b4).

#### 3.1.2 Visible success experiences and the accumulation of confidence

As practice continued, students began to experience a sense of accomplishment associated with “truly completing the movements.” Students reported that when they were able to independently perform the full routine for the first time, they experienced a marked increase in achievement and confidence. “When I finally completed the whole set without any prompts, I had a strong feeling of ‘I can really do this,’ and that feeling kept motivating me” (S04). Teachers also facilitated students’ bodily experience through somatic awareness training. “The teacher often asked us to close our eyes and feel the shift of body weight. That is usually when students begin to truly experience the movement” (T01). Students generally believed that teachers’ immediate recognition of even minor progress could continuously enhance their willingness to engage. “Even a simple comment from the teacher like ‘you look more fluent today’ can positively affect my emotional state for a long time” (S06). In addition, teachers linked Tai Chi practice to long-term health outcomes to help students understand its broader significance. “I share cases of long-term improvements in physical condition after sustained Tai Chi practice. This helps students connect the movements with future health benefits” (T05). As successful experiences accumulated, students began to proactively record practice videos and engage in self-observation and movement adjustment (a75), and their learning approach gradually shifted from reliance on external evaluation to active self-regulation.

### 3.2 Theme 2: peer internalization pathway

Theme 2 illustrates the role of peer interaction in sustaining learning engagement. Participants’ accounts indicated that peers not only provided technical assistance, but also influenced students’ attitudes toward Tai Chi and their patterns of engagement through emotional companionship, collaborative practice, and social comparison.

#### 3.2.1 Emotional companionship and behavioral influence

Students noted that practicing with peers reduced perceived pressure and increased enjoyment in the learning process. Some students spontaneously formed practice groups after class, developing stable interaction patterns through shared practice. “We formed a practice group after class, often standing unsteadily while laughing together. That shared laughter turned practice from pressure into enjoyment” (S02). Other students emphasized that sustained companionship and encouragement from peers helped them navigate periods of difficulty in learning. “My roommate kept encouraging me to go to the courtyard every night to practice. Gradually, I began to feel physical relaxation, and a kind of quiet inner calm emerged. That feeling eventually became the reason I kept practicing” (S13). Peers’ engagement behaviors also influenced others through behavioral modeling. “After class I often feel physically and mentally exhausted. Practicing Tai Chi slowly with my partner feels almost like group therapy. We supervise each other, and giving up is no longer an option” (S10). Teachers similarly observed a modeling effect among students with similar backgrounds: “When students see classmates like themselves gradually mastering the movements, many of them believe they can do it as well” (T08). However, the data also indicated that peer interaction did not always produce positive effects. When there were large gaps in ability among students, some experienced frustration due to frequent social comparison (b3). In addition, peer support that was arranged in a compulsory manner sometimes led to resistance (b2).

#### 3.2.2 Social comparison and collaborative responsibility

Participants’ accounts indicated that social comparison and collective responsibility among peers further influenced learning engagement. Some students reported that being elected as group leaders or demonstrators prompted them to voluntarily increase their practice time in order to avoid negatively affecting group performance. “As a group leader, I need to be able to demonstrate the complete set of movements. This sense of responsibility pushed me to practice on my own at night, and eventually I realized that I was no longer just imitating—I was truly understanding the movements” (S09). “Our group was selected to represent the class in a performance. During that period, we practiced at the training venue every evening because no one wanted to delay the group’s progress” (S15). Some students also noted that their understanding of movements deepened when they helped others practice. “Teaching others is often the moment when students themselves reach the deepest level of understanding” (T07). “When my peer commented that my ‘White Crane Spreads Its Wings’ looked elegant, I felt encouraged and recognized, and wanted to keep improving” (S02). In addition, some students pointed out that excessive peer correction could weaken their willingness to explore independently (b5), while social loafing within groups (b6) reduced the motivation of some members to practice consistently.

#### 3.2.3 Motivational internalization and value identification

As practice deepened, some students began to transform Tai Chi from a course requirement into a personalized health practice. Teachers helped students develop a deeper understanding of practice by explaining the breathing patterns, bodily awareness, and cultural meanings embedded in Tai Chi. “When teaching the movement ‘Grasp the Sparrow’s Tail,’ I explain how it relates to guiding qi through the meridians. When students begin to feel circulation and warmth in their hands, practice often shifts from a course requirement to a personally meaningful pursuit” (T05). Students also reported that, when facing exam pressure or daily life stress, Tai Chi practice became a form of emotional regulation. “During periods of heavy exam pressure, the breathing techniques taught by the teacher effectively calm my heartbeat and relieve anxiety. At that point, Tai Chi shifts from a university course to a personal coping resource” (S06). Some students further connected Tai Chi practice with their self-identity. “The teacher’s movements look very stable, elegant, and fluid. I also want to reach that level” (S11).

### 3.3 Theme 3: synergistic support dynamics

Theme 3 illustrates the interrelationship between teacher support and peer support in the process of learning Tai Chi. Participants’ experiences indicated that classroom activities organized by teachers often shaped the ways in which peer interactions emerged, while feedback and collaboration among peers further influenced students’ classroom experiences and their willingness to engage in sustained practice. Teacher support and peer support were generally present simultaneously and interacted with each other during the process of collective practice.

#### 3.3.1 Reciprocal reinforcement between teacher and peer support

The interaction structures designed by teachers typically provided the foundation for peer collaboration, while immediate feedback among peers further reinforced students’ engagement experiences. “After each movement, the teacher asked us to pair up and correct each other. My partner told me that I looked more relaxed than before, and suddenly I felt that maybe I could really do it” (S04). “Long theoretical explanations often made me lose focus. But when I practiced with my partner, he helped adjust my arm while saying ‘relax here.’ At that moment, I understood the movement pathway and could fully engage in it” (S07). Collective practice and group collaboration were also frequently emphasized by students as important experiences. Most students believed that group-based practice not only enhanced a sense of responsibility but also improved overall engagement. “Our group’s final performance was one of the most memorable moments of the course. The shared goal and effort really inspired everyone” (S09).

#### 3.3.2 Fragility and conditional dependence of the support system

Although teacher support and peer support can jointly promote sustained learning engagement, participants’ experiences also indicated that this support system is context-dependent to some extent. When teachers provided insufficient individual attention over time, some students began to shift toward mechanical imitation and passive participation (b4). At the same time, when peer interaction decreased after the course ended, some students’ practice habits were also interrupted. “After the final exam, no one invited me to practice anymore, and the weather also got colder. I lost motivation and stopped practicing altogether” (b11). “After the performance, there was no longer a plan for regular group practice. When the weather became colder, my own engagement started to decline” (S15). Therefore, teacher support is more closely associated with students’ competence beliefs and learning confidence, whereas peer support is more strongly related to emotional belonging, collaborative responsibility, and value identification. In actual teaching practice, teacher support and peer support typically operate jointly and together shape the formation and maintenance of sustained learning engagement among students. The thematic relationships identified in the data were integrated into a “dual-support–dual-pathway” explanatory model (Figure 1). The dual-support–dual-pathway model presented in Figure 1 clarifies two relatively independent yet mutually reinforcing psychosocial pathways: teacher support facilitates self-efficacy development through teaching empowerment, while peer support contributes to motivational internalization via horizontal peer interaction. Their synergistic interplay jointly sustains long-term Tai Chi learning engagement, marking this study’s core theoretical contribution to embodied physical education research.

**Figure 1:**
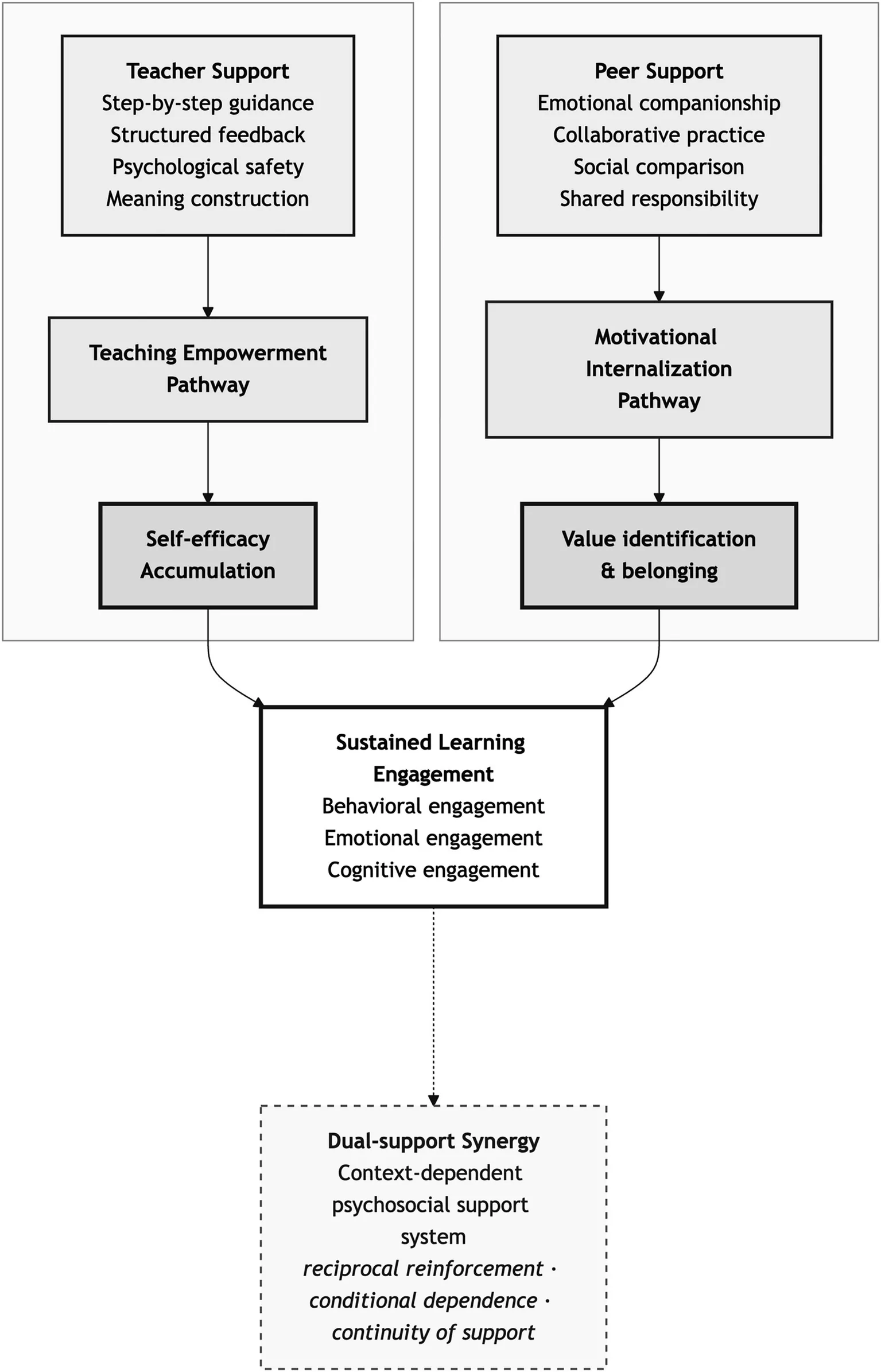
Dual-support–dual-pathway model of sustained learning engagement.

Specifically, teacher support primarily contributed to the development of self-efficacy through instructional empowerment, whereas peer support primarily facilitated motivational internalization through emotional and collaborative processes. These pathways could operate independently but were more often mutually reinforcing, jointly sustaining learning engagement over time.

## 4 Discussion

### 4.1 Teaching empowerment mechanism

Teachers constructed a “cognitive scaffold” for students through strategies such as step-by-step demonstration, task segmentation, and timely feedback, thereby lowering the learning threshold of Tai Chi as a skill-based course. The psychologically safe environment created by teachers—characterized by being “free from ridicule”—enabled students to boldly attempt movements and thus obtain valuable initial mastery experiences (e.g., independently completing a movement sequence). These observable and experiential successes constitute one of the most powerful sources of self-efficacy (11, 12). The findings further suggest that in physical skill learning contexts, teachers’ immediate recognition (e.g., “your rhythm control was very good just now”) and attention to subtle progress generate a cumulative reinforcing effect on efficacy beliefs. This aligns with the concept of comprehensibility proposed by Antonovsky (26) and Tao and Yu (9): when students can clearly understand movement requirements and perceive their own progress, they are more likely to appraise practice challenges as manageable tasks rather than burdens. In this study, teachers not only provided technical instruction but also endowed practice with cultural and health-related meaning by sharing their own learning experiences and linking Tai Chi to traditional Chinese meridian theory and stress regulation. This process of “meaning-making” corresponds with the “sense of meaning” dimension identified in university health promotion literature (27, 28). When students perceived the long-term value of practice beyond academic credit, external motivation began to be internalized. The teaching empowerment pathway indicates that the role of teachers extends beyond technical instruction to that of facilitators of meaning construction. This finding extends Zimmerman (29) and Spittle et al. (30) discussions on empowerment in health promotion: empowerment is not merely the transfer of control to individuals, but also requires structured support and clear pathways that enable individuals to genuinely develop the belief that “I can do it.” However, the study also identified boundary conditions of empowerment failure. When teachers placed overly strict emphasis on movement accuracy, timely feedback shifted into controlling correction, leading students to experience practice-related anxiety. Moreover, when teachers provided no individual attention throughout the semester, some students withdrew to the back of the class and engaged in mechanical imitation, reflecting superficial participation. These negative cases indicate that the quality of teacher support, rather than its mere presence, is critical. Both excessively rigid and entirely absent teacher support may undermine the development of self-efficacy.

### 4.2 Peer internalization mechanism

Peer support promotes the internalization of learning motivation through emotional embeddedness and social comparison. Unlike teacher support, peer support operates as a horizontal resource, encompassing emotional companionship, behavioral modeling, shared responsibility, and a sense of belonging. The spontaneously formed after-class practice groups, the responsibility-driven motivation of “not wanting to delay the group’s progress,” and the increased confidence gained from teaching peers all represent key mechanisms through which motivation shifts from “external requirement” to “internal endorsement.” From the perspective of Fredricks et al. (5), peer support enhances students’ manageability (31). When students forget movements or experience physical fatigue, peer encouragement, demonstration, or joint practice may lead them to perceive that “this problem is something I can handle.” In this study, students described that “my roommate kept encouraging me to practice in the courtyard every night; gradually I began to feel physical relaxation,” which reflects manageability in a situated context. At the same time, social comparison among peers (including parallel and upward comparisons) may either stimulate motivation or generate pressure. Boundary cases showed that when students observed peers who mastered movements significantly faster than themselves, some experienced a decline in self-efficacy. This finding suggests that the effect of peer support is not unidirectionally positive but highly dependent on the distance of comparison targets and individual psychological characteristics. Furthermore, the study demonstrates how peer support facilitates motivational internalization and identity formation. When students were elected as group leaders, demonstrators, or teaching assistants, a sense of responsibility-driven engagement emerged. This responsibility may initially stem from external expectations, but with continued practice and improvement in competence, it gradually transforms into an identity such as “I am a Tai Chi practitioner.” As one student stated: “I no longer feel that I am just completing a course; I am striving to become a person who is calm and focused.” This process is consistent with Keyes (32) concept of flourishing, which encompasses positive emotions, as well as a sense of social contribution and belonging (33). In collective Tai Chi practice and performance contexts, students experienced a sense of collective responsibility—“not wanting to let the team down”—and a shared sense of accomplishment upon completing performances, both of which reflect social contribution and collective identity. The findings also indicate that peer support plays a unique role in alleviating learning anxiety, exam stress, and interpersonal loneliness. For example, students facing academic pressure reported that “Tai Chi breathing techniques combined with peer practice were an effective form of emotional regulation.” These findings support Biddle (34), suggesting that students need to actively engage in health-promoting activities, with peer groups serving as a central component of such processes (35). However, negative cases also revealed that compulsory peer arrangements, excessive peer correction, and social loafing may weaken autonomous exploration or trigger resistance. Therefore, the structure of peer support should respect student autonomy and avoid formalistic “peer support for its own sake.”

### 4.3 Dual-support synergy pathway

The “dual-support synergy” between teacher support and peer support does not operate as two independent mechanisms; rather, it forms a positive feedback loop. Structured arrangements provided by teachers (e.g., corrective feedback and group performances) create a framework and opportunities for peer interaction. In turn, peers contribute emotional value, immediate feedback, and horizontal recognition that cannot be substituted by teachers. Participants frequently perceived this synergistic effect as more influential than either form of support operating in isolation. When teachers design activities such as “pair work,” peer feedback (e.g., “you look more relaxed this time”) is often more readily accepted by students because peers are perceived as being at a similar learning stage, and their feedback is therefore more closely aligned with students’ own experiences. When teachers organize group performances, the sense of responsibility among peers—“not wanting to delay the group’s progress”—drives autonomous after-class practice, which in turn further strengthens students’ identification with Tai Chi. When teachers share short videos of previous students’ successful performances, peer modeling effects are activated, fostering a belief of “if others can do it, I can too.” These findings indicate a significant interaction between teacher support and peer support, together constituting a “dual external resource system.” From a health promotion perspective, this aligns with Antonovsky (26) and Lee et al. (36), who emphasize a health-generating approach to university health promotion, focusing on “what promotes health” rather than “what causes illness.” In this study, teachers and peers jointly created a resource-rich environment for sustaining learning engagement: teachers provided comprehensibility (clear movement breakdowns) and meaningfulness (cultural and health-related value), while peers contributed manageability (emotional support and social comparison). The findings suggest that their combination may contribute to students’ overall sense of coherence. When students were able to understand, manage, and identify with their practice, learning engagement became less dependent on external conditions and more internalized as an autonomous and sustained practice. However, boundary cases also revealed limitations of this dual system. When teacher support was absent (e.g., no individualized attention) or peer support was interrupted (e.g., no post-course practice invitations or seasonal/weather constraints), students’ engagement declined and could even cease entirely. “After the final exam, no one invited me to practice anymore, and the weather also got colder. I lost motivation and stopped practicing altogether.” This case indicates that motivational internalization is highly dependent on a sustained social support environment. When external conditions disappear, students whose internalization is incomplete are likely to revert to non-practice. This finding has important implications for university physical education design: sustaining students’ autonomous practice beyond course completion may require establishing post-course peer communities, providing online resources, or designing progressive group performance opportunities to extend the effects of the dual-support system. The present findings are broadly consistent with international studies showing that teacher support and peer connectedness jointly contribute to engagement in higher education and physical activity settings (15, 18). However, the current study extends this literature by demonstrating how these support processes become embedded within embodied learning experiences and culturally meaningful practices such as Tai Chi.

### 4.4 Negative cases and boundary conditions

Negative and boundary cases (b1–b11) were retained during the coding process. These cases not only enhanced the credibility of the study but also provided a form of boundary testing for the proposed theoretical model. Several conditions were identified that may weaken or interrupt the normal functioning of the dual-support pathways: (1) Quality of support: When teacher support took the form of “controlling correction” (b1) or “perceived unfair praise” (b8), the original empowering effect was reversed, leading to anxiety and feelings of relative deprivation. When peer support manifested as “excessive correction” (b5) or “compulsory arrangement” (b2), students’ autonomous exploratory motivation was suppressed; (2) Sustainability of support: When the support system was disrupted (b7: no one available to consult after forgetting movements; b11: reduced motivation due to cold weather and lack of group encouragement), learning engagement declined. This suggests that the maintenance of engagement requires the continuous presence of a support system, particularly in the early stages of skill acquisition; and (3) Individual differences: Students varied in their sensitivity to different forms of support. For instance, some students were highly responsive to teachers’ non-verbal cues (smiles, nods; S16), while others relied more on verbal encouragement from peers (S05). When the form of support did not match individual preferences, its effectiveness could be limited even if support was present. These boundary conditions indicate that the proposed “dual-support–dual-pathway” model is not universally applicable across all contexts and individuals. Future research should further examine how individual differences (e.g., personality traits, prior experience, and cultural background) moderate the relationship between social support and learning engagement.

### 4.5 Limitations and future directions

This study has several limitations: (1) The research context was limited to university Tai Chi courses in China, and the findings may reflect cultural values associated with collectivism, teacher authority, and traditional health practices. Therefore, caution should be exercised when transferring the findings to different cultural settings. Tai Chi emphasizes bodily perception, breathing regulation, and long-term practice, and its learning process may differ from competitive sports such as ball games and track and field, as well as from non-sport skill-based courses. Therefore, future research could further examine the applicability and external validity of the “dual-support–dual-pathway” model in other embodied or skill-based learning contexts, such as dance, music, and foreign language learning; (2) A cross-sectional, retrospective interview design was adopted, relying primarily on participants’ recollections of prior learning experiences. Although this approach allows for rich contextual descriptions and insights into psychological change processes, it is limited in capturing the dynamic evolution of teacher support, peer support, and sustained learning engagement over time. Future studies could adopt longitudinal qualitative designs, experience sampling methods, or mixed-method approaches to track the developmental trajectories of self-efficacy and motivational internalization across different learning stages; and (3) The data were primarily based on self-reports, which may be subject to social desirability bias and limitations in subjective interpretation. Although negative and boundary cases were incorporated and constant comparison was used to enhance analytical rigor, future research could integrate multiple data sources such as classroom observations, learning journals, or physiological indicators to further improve interpretive depth and ecological validity. Despite these limitations, this study, through systematic qualitative analysis, constructed a “dual-support–dual-pathway” model explaining how teacher support and peer support promote sustained learning engagement in university Tai Chi courses, offering a new theoretical perspective for understanding social support mechanisms in skill-based embodied learning contexts. The findings also suggest that university physical education should not only focus on technical instruction itself, but also place greater emphasis on building supportive learning environments and sustaining peer interaction, thereby promoting the long-term development and maintenance of health-related engagement behaviors among students.

## 5 Conclusion

This study, based on qualitative interview data, proposes a “dual-support–dual-pathway” explanatory model to illustrate the formation mechanism of sustained learning engagement in university Tai Chi courses. The results indicate that teacher support is primarily reflected in a “teaching empowerment pathway,” whereby step-by-step guidance, structured feedback, and the creation of a psychologically safe environment help students develop perceivable mastery experiences and enhance self-efficacy. In contrast, peer support is characterized as a “motivational internalization pathway,” through which emotional companionship, collaborative practice, and social interaction gradually shift students’ course participation from external requirements toward internal identification and the formation of identity-based meaning. Both forms of support coexist within the same learning context and, as reflected in participants’ accounts, demonstrate mutually reinforcing experiential characteristics: teachers provide a structured learning framework, while peers offer emotional and interactive support, together constituting a key psychosocial foundation for sustained learning engagement. In addition, participants’ narratives suggest that this support system is context-dependent to some extent. When teacher support is insufficient or takes the form of controlling feedback, or when peer interaction is weakened due to contextual changes (e.g., course completion or social comparison pressure), students’ learning engagement may decline, indicating that both the quality and continuity of social support are critical for sustaining engagement. Based on these findings, this study provides an integrated psychosocial explanatory framework for understanding learning engagement in embodied physical education contexts, emphasizing that learning engagement emerges from the intertwined effects of teacher empowerment and peer internalization mechanisms. From a public health perspective, the findings suggest that university physical education programs should move beyond skill instruction alone and actively cultivate supportive psychosocial environments that encourage long-term participation in health-promoting physical activities. Interventions that combine structured teacher guidance with sustainable peer-support networks may help strengthen students’ lifelong engagement in active and healthy lifestyles.

## Data Availability

The original contributions presented in the study are included in the article/Supplementary material, further inquiries can be directed to the corresponding author.
