# Supplementary material for: Psychosocial mechanisms of sustained learning engagement in university Tai Chi courses: a qualitative study of teacher–peer dual-support synergy and learning pathways
Source: Front Public Health. 2026 Sep 15;14:1876692. doi: 10.3389/fpubh.2026.1876692 (PMC13619620; doi:10.3389/fpubh.2026.1876692)
Supplement: Supplementary file 1 [file Table_1.DOCX]

**Appendix S1. Teacher Participant Profiles (n = 8)**

| ID | Gender | Years of Teaching Experience | Educational Background | Discipline | Academic Rank | Background Description |
| --- | --- | --- | --- | --- | --- | --- |
| T01 | Male | 10 | Master’s Degree | Traditional Chinese Physical Education | Lecturer | Possesses a professional background in martial arts and specializes in decomposing Tai Chi movements into modular practice combinations, with emphasis on guiding students’ bodily perception. |
| T02 | Female | 18 | Bachelor’s Degree | Physical Education | Associate Professor | Has long taught general physical education courses, excels at creating a relaxed classroom atmosphere, and pays close attention to students’ emotional changes while providing individualized encouragement. |
| T03 | Male | 5 | Master’s Degree | Exercise Physiology | Teaching Assistant | A young instructor with extensive knowledge of the health benefits of Tai Chi, frequently integrating explanations of physiological and psychological mechanisms into instruction. |
| T04 | Female | 12 | Master’s Degree | Physical Education and Training Science | Lecturer | Emphasizes movement accuracy and corrective feedback, often organizing post-class group correction sessions, with a meticulous and rigorous teaching style. |
| T05 | Male | 22 | Bachelor’s Degree | Traditional Chinese Medicine | Professor | Possesses knowledge of meridian theory in Chinese medicine and frequently connects Tai Chi movements with qi and blood regulation, thereby endowing practice with deeper cultural significance. |
| T06 | Female | 7 | Master’s Degree | Psychology | Lecturer | Focuses on students’ self-efficacy and exercise motivation, and designs collaborative classroom games to reduce learning anxiety. |
| T07 | Male | 15 | Doctoral Degree | Sociology of Sport | Associate Professor | Emphasizes embodied understanding in Tai Chi rather than mere imitation, and frequently organizes peer feedback and dialogue activities. |
| T08 | Female | 3 | Master’s Degree | Martial Arts and Traditional Ethnic Sports | Teaching Assistant | A representative of the younger generation of instructors who maintains close rapport with students and effectively stimulates interest through short-video sharing and role-model examples. |

**Appendix S2. Student Participant Profiles (n = 16)**

| ID | Gender | Academic Year | Major Category | Role/Experience | Background Description |
| --- | --- | --- | --- | --- | --- |
| S01 | Male | Freshman | Engineering | None | First encountered Tai Chi after university enrollment; initially motivated by course credit requirements. Demonstrated stiff movements at the beginning and relied heavily on detailed teacher explanations. |
| S02 | Female | Freshman | Economics and Management | Class Arts and Sports Committee Member | Outgoing and sociable, enjoys group activities, often organizes dormitory peers for practice, and shows strong interest in Tai Chi culture. |
| S03 | Male | Sophomore | Agricultural Sciences | Tai Chi Club Officer | Has extensive practice experience and serves as a demonstrator in class. Frequently consulted by classmates and exhibits high self-efficacy. |
| S04 | Female | Sophomore | Medicine | None | Holds high self-expectations and initially experienced frustration when unable to memorize movements, expressing a strong need for clear step-by-step instruction from teachers. |
| S05 | Male | Sophomore | Information Technology | None | Particularly attentive to rhythm in Tai Chi practice; even brief peer encouragement regarding rhythm substantially enhanced confidence. Shows preference for technical details. |
| S06 | Female | Junior | Teacher Education | Student Union Officer | Faces pressure from teacher recruitment examinations and hopes Tai Chi can help regulate emotions. Demonstrates strong sensitivity to teacher attention and care. |
| S07 | Male | Junior | Engineering | None | Possesses strong practical ability but easily loses focus during theoretical explanations. Demonstrates greater concentration during paired peer practice. |
| S08 | Female | Freshman | Arts | Psychological Committee Member | Sensitive about bodily coordination, and emotionally influenced by teachers’ gentle corrective approaches. |
| S09 | Male | Sophomore | Mechanical Engineering | Group Leader | Developed confidence through leading group practice, and showed significantly increased willingness to practice after receiving peer praise for standardized movements. |
| S10 | Female | Junior | Economics and Management | Intern | Began practicing Tai Chi to alleviate employment anxiety and gained a sense of group belonging through collective practice. |
| S11 | Male | Freshman | Automotive Engineering | None | Regards the course instructor as a traditional martial arts role model and frequently imitates the teacher’s movement style as motivation for continued practice. |
| S12 | Female | Junior | Information Technology | Peer Teaching Assistant | Previously served as a senior student teaching assistant; strengthened her own self-efficacy and persistence through guiding junior students. |
| S13 | Male | Sophomore | Medicine | None | Initially resistant due to course reassignment, but gradually experienced bodily relaxation and enjoyment through peer companionship. |
| S14 | Female | Freshman | Agriculture and Forestry | Dormitory Life Committee Member | Introverted personality; gradually joined the practice circle under the influence of roommates, transitioning from observer to active participant. |
| S15 | Male | Junior | Civil Engineering | Club Leader | Places high importance on practice outcomes and demonstrates strong engagement while leading peers in rehearsing collective Tai Chi performances. |
| S16 | Female | Sophomore | Arts | None | Highly sensitive to teachers’ nonverbal approval (e.g., smiles and nods), and positive feedback effectively alleviates feelings of monotony. |

**Appendix S3. Semi-Structured Interview Protocol**

| Core Dimension | Participants | Sample Key Questions | Purpose of Design |
| --- | --- | --- | --- |
| Teacher Support | Teachers | How do you help students with poor coordination build confidence? Have there been cases in which guidance failed? | To explore structured support and its boundary conditions |
| Teacher Support | Students | Describe a moment when a teacher made you feel that “I can learn this.” Have you had any negative experiences? | To capture perceptions of support and its effectiveness |
| Peer Support | Teachers | How do you perceive peer interaction during group practice? Have you observed any negative peer influences? | To understand teachers’ observations of peer systems |
| Peer Support | Students | How have peers helped you overcome difficulties in practice? Have you experienced negative comparison? | To examine emotional and behavioral peer effects |
| Self-Efficacy | Teachers and Students | At what moment did students demonstrate a clear increase in confidence? What causes persistently low self-efficacy? | To identify key events and barriers in efficacy construction |
| Learning Motivation | Teachers and Students | How does motivation shift from credit-oriented participation to interest-driven engagement? In what situations does motivation decline? | To capture the process and obstacles of motivational internalization |
| Learning Engagement | Teachers and Students | What signs indicate that students have entered a state of deep engagement? What are common causes of practice interruption? | To explore explicit indicators of engagement and withdrawal |

**Appendix S4. Initial Concepts from Open Coding (n = 86)**

**S4-1 Positive Concepts (a1–a75)**

| ID | Initial Concept |
| --- | --- |
| a1 | Teachers’ step-by-step demonstrations lower the threshold for learning movements |
| a2 | Imitating teachers’ movements generates an initial sense of achievement |
| a3 | Teachers establish flexible practice standards to protect students’ self-esteem |
| a4 | Protective corrective feedback reduces frustration |
| a5 | Practicing alongside peers generates shared enjoyment |
| a6 | Teachers intentionally design peer-assisted practice structures |
| a7 | Spontaneous formation of after-class Tai Chi practice groups |
| a8 | Shared interests foster an atmosphere of mutual learning |
| a9 | Senior students guide junior students by transmitting practice experience |
| a10 | Emotional companionship from peers supports students through bottlenecks |
| a11 | Observing peers’ progress stimulates persistence |
| a12 | Seeing similar peers master movements inspires engagement |
| a13 | Successfully performing an entire routine independently establishes mastery |
| a14 | Praise from peers directly enhances confidence |
| a15 | Collaborative performances generate sensitivity to evaluation |
| a16 | Concern about peer evaluation becomes an external driving force |
| a17 | Teachers arrange task sequences beginning with simple movements |
| a18 | Successful experiences restore confidence in one’s abilities |
| a19 | Increased confidence encourages attempts at more difficult movements |
| a20 | Verbal comfort from peers alleviates practice anxiety |
| a21 | Emotional recovery facilitates re-engagement in practice |
| a22 | Public recognition from teachers enhances motivation |
| a23 | Peer trust increases willingness to think actively during practice |
| a24 | Serving as a group leader generates responsibility-driven engagement |
| a25 | Stable peer relationships provide sustained practice environments |
| a26 | Internalizing peers’ persistence as self-expectation |
| a27 | Collective group progress enhances efficacy |
| a28 | Avoiding letting down the team motivates additional private practice |
| a29 | Teachers articulate long-term health visions associated with practice |
| a30 | Ambiguous course goals become concrete and understandable |
| a31 | Clarity generates immediate impulses to practice |
| a32 | Peers introduce extracurricular Tai Chi resources |
| a33 | Peers’ diligence influences one’s own behavior |
| a34 | Self-practice volume adjusted according to peer standards |
| a35 | High sensitivity to subtle teacher facial expressions |
| a36 | Immediate comfort from teachers instantly repairs self-efficacy |
| a37 | Feedback regulates bodily tension and emotional distress |
| a38 | Being required to serve as a demonstrator activates hidden confidence |
| a39 | Successful end-of-class group performances create peaks of confidence |
| a40 | Feelings of bodily lightness and comfort enhance efficacy |
| a41 | Teachers sharing their own beginner experiences builds relational trust |
| a42 | Teachers connect practice with stress-regulation meaning |
| a43 | Sense of meaning sustains practice beyond course credit requirements |
| a44 | Teachers create a safe environment free from ridicule |
| a45 | Safety encourages trial and error without fear of embarrassment |
| a46 | Safety fosters preliminary identity identification with Tai Chi |
| a47 | Teaching movements to peers deepens understanding and confidence |
| a48 | Repeated successful practice stabilizes efficacy beliefs |
| a49 | High-performing peers generate upward comparison pressure |
| a50 | Peer competence invisibly raises self-expectations |
| a51 | Peers’ enthusiasm directly motivates observers to participate |
| a52 | The first perfect performance activates intrinsic interest |
| a53 | Shared experiences overcoming hot weather create emotional bonds |
| a54 | Group practice simulates collaborative working roles |
| a55 | Teachers’ smiles and nods function as silent reinforcement |
| a56 | Feeling noticed by teachers generates enjoyment in practice |
| a57 | A fluent demonstration overturns peers’ previous underestimation |
| a58 | Being admired significantly enhances self-worth |
| a59 | The bodily sensation of a perfect Tai Chi performance becomes a lasting memory |
| a60 | Remote peer-organized practice during internships alleviates occupational stress |
| a61 | Shared experiences of forgetting movements foster mutual understanding |
| a62 | Small acts of peer care (e.g., offering water or reserving space) restore engagement |
| a63 | Teachers are internalized as role models of physical and mental well-being |
| a64 | Peers evolve into long-term exercise partners |
| a65 | Shared visions for collective performance strengthen engagement |
| a66 | Mutual movement correction improves collaborative technical skills |
| a67 | Shared persistence cultivates group attachment |
| a68 | Tai Chi enhances perceptions of bodily control |
| a69 | Teachers’ cultural explanations imbue movements with philosophical meaning |
| a70 | Music-assisted practice enhances concentration and immersion |
| a71 | Tai Chi breathing techniques are applied to pre-examination anxiety regulation |
| a72 | Experiencing bodily relaxation and calmness becomes an intrinsic driver |
| a73 | Teachers’ immediate acknowledgment of minor progress |
| a74 | Visible progress from stiffness to fluidity |
| a75 | Active recording of practice videos for self-comparison and optimization |

**S4-2 Negative and Boundary Concepts (b1–b11)**

| ID | Initial Concept | Type |
| --- | --- | --- |
| b1 | Excessive teacher demands for movement accuracy generate fear of practice | Negative case |
| b2 | Forced peer-assistance arrangements trigger resistance among some students | Negative case |
| b3 | Observing peers master movements too quickly reduces self-efficacy | Boundary case |
| b4 | Lack of individual teacher attention results in superficial engagement | Negative case |
| b5 | Peers’ excessive correction undermines autonomous exploration motivation | Negative case |
| b6 | Social loafing within groups weakens willingness to engage | Boundary case |
| b7 | Practice interruption occurs when no peers are available for consultation after forgetting routines | Negative case |
| b8 | Teachers praising only a few outstanding students generates relative deprivation among others | Negative case |
| b9 | Social media displays of advanced skills intensify self-doubt | Boundary case |
| b10 | Lack of phased goals creates feelings of stagnation and disengagement | Boundary case |
| b11 | Cold weather and lack of collective encouragement terminate morning practice routines | Negative case |

**Appendix S5. Axial Coding: Five Core Categories**

**Core Category 1: Teacher-Structured Empowerment**

| Related Initial Concepts | Category Definition |
| --- | --- |
| a1, a3, a4, a6, a17, a22, a29, a41, a42, a44, a45, a55, a56, a63, a69, a73 | Teachers construct cognitive scaffolding and psychologically safe learning environments through stepwise task arrangement, protective feedback, cultural interpretation, and role-modeling. |
| Boundary Tensions: b1 (controlling correction); b4 (absence of attention); b8 (inequitable praise) | Support pathways become disrupted when support is perceived as controlling, unfair, or persistently absent. |

**Core Category 2: Peer-Embedded Collaboration**

| Related Initial Concepts | Category Definition |
| --- | --- |
| a5, a7, a8, a9, a10, a15, a16, a20, a23, a25, a26, a32, a33, a34, a49, a50, a51, a53, a54, a57, a58, a60, a61, a62, a64, a66, a67 | Peers become embedded within the learning process through emotional companionship, behavioral contagion, social comparison, and collaboration, thereby providing belongingness and normative references. |
| Boundary Tensions: b2 (forced assistance); b3 (upward comparison reducing efficacy); b5 (overdependence on peer correction); b6 (social loafing); b7 (interruption of peer accessibility) | Peer interactions lacking autonomy or involving excessive disparities may generate counterproductive effects, weaken autonomy, and undermine engagement. |

**Core Category 3: Accumulative Construction of Self-Efficacy**

| Related Initial Concepts | Category Definition |
| --- | --- |
| a11, a12, a13, a14, a18, a19, a38, a39, a40, a47, a48, a59, a68, a72, a74 | Self-efficacy is cumulatively constructed through direct success experiences, peer recognition, improved bodily perception, and role enactment, ultimately forming stable efficacy beliefs. |
| Boundary Tensions: b3 (comparison-induced efficacy decline); b9 (social media performance pressure); b10 (lack of progress perception) | Self-efficacy may enter a downward spiral when students fail to perceive progress signals or repeatedly encounter unfavorable comparisons. |

**Core Category 4: Motivational Internalization and Value Identification**

| Related Initial Concepts | Category Definition |
| --- | --- |
| a2, a24, a27, a30, a31, a43, a46, a52, a65, a71 | External requirements gradually transform into health identification, self-cultivation pursuits, and identity recognition, thereby forming autonomous motivation that persists beyond course credit requirements. |
| Boundary Tensions: b10 (absence of goals); b11 (practice cessation without group support) | When internalization remains incomplete, motivation rapidly declines once external conditions disappear, revealing the fragility of insufficient internalization. |

**Core Category 5: Manifestations of Sustained Engagement**

| Related Initial Concepts | Category Definition |
| --- | --- |
| a21, a28, a36, a37, a53, a62, a70, a75 | Engagement is manifested through re-engagement following emotional recovery, additional time investment, embodied immersion, active self-monitoring, and strategic self-optimization. |
| Boundary Tensions: b7 (practice interruption due to unavailable support); b11 (environmentally induced disengagement) | Engagement is not permanently stable and may diminish whenever support systems become disrupted, reflecting its processual and context-dependent nature. |

**Appendix S6. Selective Coding: Three Integrative Themes**

*Theme 1: Instructional Empowerment Pathway — From Structured Support to Efficacy Conviction*

Core Categories: Teacher-Structured Empowerment → Accumulative Construction of Self-Efficacy → Manifestations of Sustained Engagement

Key Concepts: a1, a17, a29, a44, a63, a13, a18, a48, a59, a19, a31, a46

Core Narrative: The cognitive scaffolding and safe atmosphere provided by teachers enable students to achieve perceivable success experiences, which progressively accumulate into efficacy beliefs of “I can do this,” ultimately leading to sustained engagement.

*Theme 2: Peer Internalization Pathway — From Emotional Embeddedness to Identity-Driven Engagement*

Core Categories: Peer-Embedded Collaboration → Motivational Internalization and Value Identification → Manifestations of Sustained Engagement

Key Concepts: a5, a20, a25, a33, a49, a67, a2, a24, a43, a52, a65, a21, a27, a38, a54

Core Narrative: The sense of belonging and emotional regulation fostered by peers gradually transforms practice motivation from externally regulated participation into internally endorsed identification, thereby generating stable autonomous engagement.

*Theme 3: Dual-Spiral Synergistic Pathway — Cross-Reinforcement Between Efficacy and Motivation*

Core Category Interaction: Teacher Empowerment Pathway × Peer Internalization Pathway

Key Concepts: a6, a9, a15, a39, a47, a57, a28, a53, a65, b4, b7

Core Narrative: Teachers provide structural guidance, whereas peers contribute emotional momentum. Together, they constitute a positive feedback loop between efficacy and motivation. The absence or mismatch of either component may destabilize the entire system.

**Appendix S7. Theoretical Model: The “Dual-Support–Dual-Pathway” Model of Learning Engagement Generation**

*Core Proposition:*
Within university Tai Chi courses, teacher support and peer support jointly constitute a dual external resource system. The teacher-support pathway primarily stimulates self-efficacy through structured empowerment, whereas the peer-support pathway promotes motivational internalization through emotional embeddedness. These two pathways function both independently and interactively, jointly facilitating the formation and maintenance of sustained learning engagement.

*Model Description:*
Teacher Support → Accumulative Self-Efficacy → Learning Engagement
Peer Support → Motivational Internalization → Learning Engagement

Additionally, a reciprocal positive loop exists between self-efficacy and motivational internalization, alongside synergistic interaction between teacher support and peer support.

**Appendix S8. Evidence Chain (Illustrative Excerpts)**

| ID | Quotation | Type |
| --- | --- | --- |
| T01 | “I break one movement into three smaller sections. Once students realize they can follow the first section, they gain confidence for the second.” | Positive |
| T02 | “Sometimes I can see that her movements are acceptable, but she is too tense. I walk over, lightly pat her shoulder, and say, ‘You’re doing well, don’t rush.’” | Positive |
| S04 | “After each movement, the teacher asked us to correct each other in pairs. My partner told me that this time I looked more relaxed than before, and suddenly I felt that maybe I really could do it.” | Positive |
| S07 | “My roommate kept saying, ‘If you don’t practice, you’ll fail the final exam.’ The more I heard it, the more irritated I became, and eventually I just wanted to avoid practice.” | Negative |
| S13 | “When I saw the person beside me performing so smoothly while I could barely maintain my stance, I gradually lost motivation.” | Boundary |
| S15 | “Our group was selected to represent the class in a performance. For those two weeks, we practiced voluntarily every evening in the square because no one wanted to let the team down.” | Positive |
| b4 | Student: “Throughout the entire semester, the teacher never spoke to me individually once, so I just stood in the last row and casually mimicked the movements.” | Negative |
| b11 | “After the final assessment, no one invited me to practice anymore, the weather became cold, and I lost motivation, so I never practiced again.” | Boundary |

**Appendix S9. Reflexivity Statement**

*Researcher Positionality*

The researcher possesses prior experience in Tai Chi practice and has observed university physical education teaching contexts, resulting in heightened sensitivity to instructional interaction processes. Although familiarity with the context may deepen interpretive understanding, it may also create risks of overinterpreting positive effects.

*Bias Management*

Reflexive journals were maintained throughout the research process to document researchers’ prior assumptions and emotional reactions. Coding was conducted independently by two researchers and subsequently discussed until consensus was achieved. During interviews, deliberate probing of negative and deviant cases was employed to avoid selectively privileging data that merely supported prior assumptions.

*Ethical Considerations*

All participants signed informed consent forms prior to participation. Personal identifying information was removed, and all participants were represented only through coded identifiers. Participants were informed that they could withdraw from the study at any time without any consequences for course grades or academic evaluation.

**Appendix S10. Teacher Participants’ English Statements and Conceptual Associations (n = 8)**

| ID | Refined Academic Statements | Associated Concepts (Codes) |
| --- | --- | --- |
| T01 | “I deconstruct each Tai Chi form into a series of micro-modules. Once students are able to complete the first segment fluently, they immediately begin to perceive the entire routine as achievable.” “I often ask students to close their eyes and attend to the transfer of body weight; this is typically the moment when they begin to internalize the movement through embodied perception.” “I intentionally provide explicit recognition for subtle improvements, such as saying, ‘Your rhythm was especially well controlled just now.’” | a1, a17, a13, a40, a68, a73, a55 |
| T02 | “I pay close attention to students who unconsciously hold their breath during practice. A gentle touch on the shoulder accompanied by a quiet reminder such as ‘Take your time’ can often release their tension within seconds.” “I avoid singling students out for mistakes. Instead, I use collective prompts such as, ‘Let us all soften our knees together,’ which helps preserve a relaxed classroom atmosphere.” “When a student finally releases tension in the shoulders, I respond with a warm nod, and their facial expression immediately brightens.” | a36, a56, a44, a4, a55 |
| T03 | “I explain how slow and deep breathing may reduce physiological stress responses. Once students genuinely experience a sense of calmness, their motivation often shifts from ‘I have to participate’ to ‘I want to experience that feeling again.’” “I incorporate partner-mirroring exercises into practice sessions. Through synchronizing breathing and movement, students often transform anxiety into interpersonal connection and shared enjoyment.” | a42, a52, a6, a66, a51 |
| T04 | “After class, I frequently remain with small groups to refine postural details. Students often correct one another, and in many cases peer explanations are understood more immediately than teacher instruction.” “I structure each session around the mastery of a single micro-skill, such as the ‘cat step.’ Each small success functions as a foundational building block that gradually accumulates into broader competence.” | a6, a47, a66, a17, a48 |
| T05 | “When teaching movements such as ‘Grasp the Sparrow’s Tail,’ I explain how the sequence is traditionally associated with guiding qi through the meridians. Once students perceive warmth or bodily flow in their hands, practice often evolves from a course requirement into a personally meaningful ritual.” “I also share examples of older adults who alleviated chronic joint discomfort through long-term Tai Chi practice. Such narratives help students connect present effort with long-term health value.” | a69, a43, a29, a63, a46 |
| T06 | “I design mirroring activities in which there is intentionally no absolute standard of right or wrong. This significantly reduces fear of evaluation and encourages students to experiment more openly.” “I frequently acknowledge students’ effort by saying things such as, ‘I noticed how focused you were on maintaining balance today.’ Recognizing effort rather than correctness alone appears to strengthen intrinsic motivational drive.” | a44, a51, a73, a22 |
| T07 | “I ask paired students to describe to one another what the sensation of ‘sung’ (release and relaxation) feels like in the hips. Such peer dialogue transforms an abstract instruction into an embodied and personally meaningful discovery.” “When I observe one student helping another adjust posture, I intentionally step back. Teaching others is often the moment when students’ own mastery deepens most substantially.” “Rather than over-correcting movements, I quietly ask, ‘How did that feel?’ This type of questioning encourages bodily awareness and supports the development of learner autonomy.” | a7, a32, a47, a4, a18, a40 |
| T08 | “During the initial weeks of the course, I present short videos of former students who began with similarly rigid movements. Observing comparable others succeed often inspires students and increases their confidence.” “We maintain a class communication group in which students upload practice videos. Peer approval through comments and encouragement frequently becomes a powerful source of sustained motivation.” | a12, a18, a51, a25, a33, a75 |

**Appendix S11. Student Participants’ English Statements and Conceptual Associations (n = 16)**

| ID | Refined Academic Statements | Associated Concepts (Codes) |
| --- | --- | --- |
| S01 | “At the beginning, I could not remember even four movements. The teacher deconstructed the routine so clearly that once I successfully repeated the first segment, I began to think, ‘Perhaps I really can learn this.’” “The teacher’s calm tone and patient step-by-step guidance created a sense of psychological safety that allowed me to appear awkward without embarrassment.” “The moment I correctly completed the opening sequence, I started genuinely looking forward to the next class.” | a1, a13, a18, a44, a52 |
| S02 | “We formed a small practice group after class and often laughed together through our unstable movements. That shared laughter transformed practice from pressure into enjoyment.” “When my friend commented that my ‘White Crane Spreads Its Wings’ movement looked elegant, I felt deeply encouraged and wanted to return the next day to improve even further.” “My motivation gradually shifted from merely earning course credits to sharing a healthy lifestyle with my social circle; Tai Chi eventually became part of our everyday interaction.” | a7, a53, a14, a58, a43, a65 |
| S03 | “Because classmates often ask me for guidance, I feel responsible for never attending practice unprepared. Teaching others compels me to refine every technical detail.” “When I compared my current practice videos with those from the first month, I could clearly see the transition from rigidity to fluidity. That visible progress strengthened my belief in the value of persistent effort.” | a47, a24, a28, a74, a48 |
| S04 | “I would sometimes forget movements midway through a routine and feel like leaving the practice space altogether. However, the teacher told me, ‘If you can master just this one section today, that is already a success.’ That small and achievable goal helped pull me out of panic.” “When I finally completed the entire routine smoothly without prompts, I experienced a powerful sense of ‘I really can do this,’ and that vivid feeling continues to motivate me.” | a3, a17, a36, a13, a59 |
| S05 | “I am particularly sensitive to rhythm. When my practice partner said, ‘You finally synchronized with the music,’ that single comment motivated me for weeks afterward.” “I gradually began to perceive Tai Chi as similar to debugging code within my own body. I started recording practice videos to examine alignment and movement details, and the process of improving frame by frame became highly engaging.” “Sometimes I watch highly polished performances online and begin doubting my own progress. However, when I compare them with my earlier recordings, I can still recognize how far I have progressed.” | a14, a22, a68, a75, a70, b9 |
| S06 | “During periods of intense examination stress, the breathing techniques taught by the instructor were the only methods that effectively calmed my racing heartbeat. At that moment, Tai Chi transformed from a university course into a personal coping resource.” “I depended heavily on acknowledgment from the teacher. Even a simple comment such as ‘You appear more centered today’ could positively influence my emotional state for an entire week.” | a71, a42, a56, a35, a63 |
| S07 | “Extended theoretical explanations often caused me to lose concentration. However, when practicing with a partner who physically adjusted my arm while saying, ‘Relax here,’ I understood the movement immediately and became fully engaged.” “Without that kind of hands-on peer support, I probably would have gradually disengaged from the course. Peer interaction was what sustained my participation.” | a5, a66, a20, a32, a25 |
| S08 | “I am highly conscious of appearing physically uncoordinated. The teacher would quietly pass by and softly say, ‘Relax your knees,’ without any tone of judgment. That gentle approach prevented me from feeling hopeless.” “Even a slight smile from the teacher after I had made a serious effort could immediately dissolve the frustration I had accumulated.” | a35, a55, a44, a56, a36 |
| S09 | “As the group leader, I needed to demonstrate the full routine competently. That sense of responsibility pushed me to practice privately at night, and eventually I realized I was no longer pretending—I genuinely understood the movements.” “When teammates told me, ‘You explain the movements very clearly,’ I experienced a strong sense of pride that further consolidated my own mastery. Our group’s final performance remains one of the most memorable moments of the course.” | a38, a24, a28, a47, a58, a39, a27 |
| S10 | “After finishing part-time work, I often felt physically and emotionally exhausted. Practicing slow Tai Chi with my partner felt almost like group therapy. We held each other accountable, and skipping practice gradually stopped feeling like an option.” “When the weather became colder and my schedule changed, my practice frequency declined. However, a single message in the group chat was often enough to encourage me to return.” | a60, a53, a67, a25, b11 |
| S11 | “My teacher’s movements appeared deeply grounded and serene. I wanted to embody that same presence, so I even began imitating the way he walked.” “That admiration gradually changed my mindset. I no longer felt that I was simply completing a course; instead, I was trying to become a calmer and more centered person like him.” | a1, a2, a63, a46 |
| S12 | “Teaching junior students made me realize how much I truly understood about the practice. Watching them finally grasp a movement became deeply rewarding for me.” “The moment they praised my teaching ability, my confidence became firmly established. I shifted from doubting my own competence to believing that Tai Chi would remain a lifelong practice for me.” | a47, a18, a48, a19, a46 |
| S13 | “At first, I strongly disliked this course because I had enrolled only due to scheduling constraints. Watching classmates master the movements quickly made me feel even more inadequate.” “However, my roommate consistently encouraged me to practice in the courtyard every evening. Gradually, I began to feel my body relax, and a quiet sense of inner calm emerged. That feeling eventually became the reason I continued practicing.” | a10, a20, a72, a52, b3 |
| S14 | “I was initially too shy to join the practice circle. My roommate simply said, ‘Let’s follow the practice video together in the dormitory.’ That small and non-pressuring invitation became the bridge I needed.” “One month later, I found myself encouraging others to practice as well. That transformation in myself was the most unexpected outcome.” | a7, a62, a19, a38 |
| S15 | “While preparing for the collective performance, we gathered in the square every evening to practice under the streetlights. None of us wanted to disappoint the group.” “That shared goal transformed us into a highly cohesive unit. The collective excitement we experienced after the final performance made all the physical fatigue worthwhile.” “After the performance ended, and without the routine of daily group practice, my own engagement declined once the weather became colder.” | a65, a28, a27, a39, a24, b11 |
| S16 | “I paid extremely close attention to the teacher’s facial expressions. When I finally performed a posture correctly and she responded with a subtle nod, it felt like emotional fuel that motivated me to continue practicing for another hour.” “However, whenever I felt invisible—when the teacher corrected everyone around me but overlooked me—I tended to withdraw to the back row and mechanically imitate the movements without genuine engagement.” | a35, a55, a56, b4 |
